# Modulating crossover positioning by introducing large structural changes in chromosomes

**DOI:** 10.1186/s12864-015-1276-z

**Published:** 2015-02-15

**Authors:** Antoine Ederveen, Yuching Lai, Marc A van Driel, Tom Gerats, Janny L Peters

**Affiliations:** Department of Molecular Plant Physiology, Radboud University Nijmegen, Institute for Water and Wetland Research (IWWR), Heyendaalseweg 135, 6525 AJ Nijmegen, The Netherlands; Netherlands Bioinformatics Centre, 260 NBIC, P.O. Box 9101, 6500 HB Nijmegen, The Netherlands; The Delft Bioinformatics Lab, Department of Intelligent Systems, Delft University of Technology, Mekelweg 4, 2628 CD Delft, The Netherlands; Current affiliation: Philips Research, High Tech Campus 11, 5656 AE Eindhoven, The Netherlands

**Keywords:** Arabidopsis thaliana, Meiosis, Crossover positioning, Recombination frequency, Chromosome modifications, Deletion, Inversion, Gamma-irradiation

## Abstract

**Background:**

Crossing over assures the correct segregation of the homologous chromosomes to both poles of the dividing meiocyte. This exchange of DNA creates new allelic combinations thus increasing the genetic variation present in offspring. Crossovers are not uniformly distributed along chromosomes; rather there are preferred locations where they may take place. The positioning of crossovers is known to be influenced by both exogenous and endogenous factors as well as structural features inherent to the chromosome itself. We have introduced large structural changes into Arabidopsis chromosomes and report their effects on crossover positioning.

**Results:**

The introduction of large deletions and putative inversions silenced recombination over the length of the structural change. In the majority of cases analyzed, the total recombination frequency over the chromosomes was unchanged. The loss of crossovers at the sites of structural change was compensated for by increases in recombination frequencies elsewhere on the chromosomes, mostly in single intervals of one to three megabases in size. Interestingly, two independent cases of induced structural changes in the same chromosomal interval were found on both chromosomes 1 and 2. In both cases, compensatory increases in recombination frequencies were of similar strength and took place in the same chromosome region. In contrast, deletions in chromosome arms carrying the nucleolar organizing region did not change recombination frequencies in the remainder of those chromosomes.

**Conclusions:**

When taken together, these observations show that changes in the physical structure of the chromosome can have large effects on the positioning of COs within that chromosome. Moreover, different reactions to induced structural changes are observed between and within chromosomes. However, the similarity in reaction observed when looking at chromosomes carrying similar changes suggests a direct causal relation between induced change and observed reaction.

**Electronic supplementary material:**

The online version of this article (doi:10.1186/s12864-015-1276-z) contains supplementary material, which is available to authorized users.

## Background

Crossing over (CO) between homologous chromosomes is actively promoted during meiosis in all but a few eukaryotic species. The establishment of a physical connection between both homologues ensures their correct segregation to both poles of the dividing meiocyte. Furthermore this exchange reshuffles the genetic deck of the parent individual, allowing new, favorable allelic combinations to arise. Large advances in our knowledge of recombination have been made in the past few decades and extensive reviews on the current state of affairs in *Arabidopsis thaliana* have recently been published [1,2]. The increasing knowledge in this area offers the possibility of exercising a degree of control over CO positioning or -frequency which holds the promise of speeding up current breeding programs.

The positioning of COs along Arabidopsis chromosomes is not uniform; rather it exhibits a pattern of cold- and hotspots where recombination frequencies can vary from 0 cM/Mb to over 85 cM/Mb in 20 kb intervals, which is more than 18 times the chromosomal average of 4.6 cM/Mb [3]. In female meiosis recombination is most proliferate around the centromere and decreases in strength towards the telomeres while in male meiosis recombination frequencies are highest in the sub-telomeric regions, minimal in the middle of chromosome arms and above average around the centromere [4].

Factors that influence CO position and hence local recombination frequency include features inherent to the chromosome as well as environmental factors of both endogenous and exogenous origin.

Exogenous environmental factors are found to severely influence the recombination landscape. Heat stress affects the transcript levels of meiotic proteins as well as their affinity for substrates [5]. High concentrations of heavy metals in Arabidopsis growth media have been reported to strongly influence rates of homologous recombination, either by directly interfering with DNA repair and replication processes or through the generation of Radical Oxygen Species (ROS) [6] that damage the DNA. Elevated levels of ROS can also occur when the efficiency of the ROS scavenging machinery is reduced following the activation of the plants pathogen defense system or through exposure to heightened levels of ionizing radiation [7]. Increased levels of DNA damage during meiosis add to the number of Double stranded DNA Breaks (DSBs) induced endogenously by SPO11 and can thus influence the number and location of COs.

Endogenous factors that have been found to directly influence CO frequencies include the evolutionary strongly conserved meiosis-specific helicase SPO11 that actively introduces DSBs in DNA the repair of which may result in CO [8]; recombination modulator *Rm1* that effects differential modification of genome-wide recombination in male and female meiocytes of *Petunia hybrida* [9] and the DNA helicase FANCM that limits the number of COs in Arabidopsis to an apparent evolutionary optimum while a *null* mutation elevates recombination levels up to four times that of controls [10].

Whereas attempts to correlate the Arabidopsis landscape of recombination to a number of chromosomal features including GC content, CpG islands, density of transposable elements and gene density seemed not to provide conclusive results [4], more recently Arabidopsis CO occurrence has been found to increase towards gene promoters and terminators whereas hotspots are found to correlate with structural features of chromosomes including H2A.Z, low nucleosome density, low DNA methylation and tri-methylation of H3K4 [11] (for an extensive review on the determinants of DSB formation see [2]). Moreover in mouse, specific sequence motifs have been identified that sequester the DSB formation machinery away from functional genomic elements through the activity of the zinc-finger protein PRDM9 [12].

Changes to the structural integrity of chromosomes have been found to drastically alter the recombination landscape in several organisms. In the yeast *Saccharomyces cerevisiae*, recombination rates of bisected chromosomes were increased compared to controls while those of fusion chromosomes were reduced [13,14]. Similar research in *Petunia hybrida* has shown incremental deletions on the short arm of chromosome VI, generated through γ-irradiation, to affect meiotic recombination between two tightly linked markers on the distal part of the long arm of that same chromosome. As the sizes of the induced deletions increased, recombination between two tightly-linked phenotypic markers increased from 5% to 50% while chromosome-wide recombination varied little from the control situation [15]. When wild-type structure was reconstituted through recombination these chromosomes regained their original recombination frequencies, showing the heightened RF to have resulted from the changed structure of the chromosome rather than from specific genes having been hit. The gradual increase of recombination with increasing deletion size combined with the stability of the total recombination rate implies a direct correlation between the length of that chromosome and the positioning of its COs. Moreover, the disproportional increase in recombination observed in the region studied suggests not an even increase in recombination events along the chromosome but rather a modification of a pattern of hot- and coldspots. Hence it has been proposed that the modification of the recombinationally active length of chromosomes, either through the introduction of deletions, inversions, translocations or homoeologous sequences, can affect CO-positioning and -frequency [16]. Exactly those types of structural changes to chromosomes have long been reported to result from exposure to ionizing radiation [17-19].

In this paper we describe the generation of chromosome-deletion and putative inversion mutants in *Arabidopsis thaliana*. Both deletions and inversions do not provide a suitable template for CO-initiation and are recombinationally silent. We examined whether chromosome-wide recombination frequencies are maintained at control levels in these mutants and if so, whether any compensation in RF is exercised locally within chromosomes, over the whole affected chromosome or genome-wide.

## Results

### γ-ray-induced changes in chromosome structure

**Figure 1:**
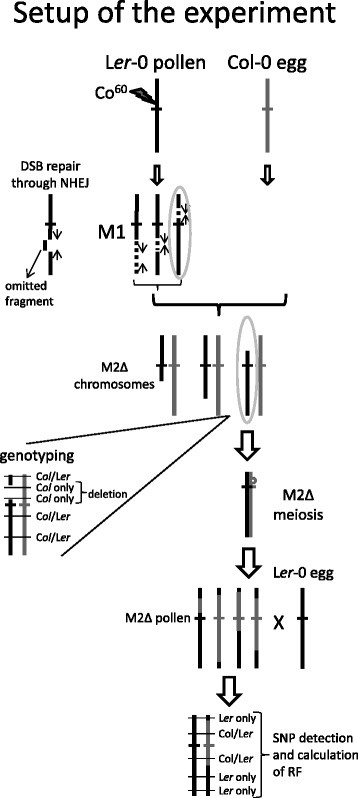
**Crossing scheme of the experiment.** Flowering individuals of *Arabidopsis thaliana* ecotype L*er*-0 were subjected to γ-irradiation to induce Double Stranded DNA Breaks (DSBs). Four groups of plants were irradiated at 0 (Controls), 150, 300 and 600 Gray. Repair of DSBs resulted in the omission or inversion of interstitial fragments, so creating large chromosomal deletions and putative inversions in the gametes of these plants (M1 generation). Crossing of these plants to ecotype Col-0 was performed on the same day. The resulting offspring (M2) were analyzed on L*er*-0 and Col-0 SNP presence at 2100 markers spread over the genome. Regions of Loss of Heterozygosity (LOH) indicating deletions (Δ) were readily observed and 16 promising M2Δ individuals carrying deletions of varying sizes spread over the five chromosomes were backcrossed to ecotype L*er*-0 for recombination analyses following SNP detection.

To study the effects of large structural changes in chromosomes on the distribution of COs during male meiosis within those chromosomes, mature pollen of *Arabidopsis thaliana* ecotype L*er*-0 was irradiated with γ-rays emitted from a Cobalt^60^ source at 150, 300 and 600 Gray. Within eight hours of irradiation the treated pollen (M1) was used to fertilize Col-0 plants. Of each resulting group of seeds (M2), ~50 were grown, their DNA was collected and subjected to Illumina GA2-based SNP-detection for deletion identification and their pollen was used to backcross to the L*er*-0 ecotype for later recombination analysis (Figure 1). Chromosomal deletions, inversions and translocations were expected to be readily induced in the pollen as a result of the three irradiation treatments.

Within the ~150 M2 individuals, 47 individuals carrying one or more chromosomal deletions (M2Δ) were identified through bioinformatic analysis of the Illumina data. Ten of these individuals displayed large areas on all chromosomes where few or no reads were mapped, making reliable sizing of their deletions equivocal. In two of these individuals putative deletions were later confirmed but the other eight were excluded from further analysis. Of the 39 remaining M2Δ individuals, 11 resulted from 150 Gray irradiated pollen; six from 300 Gray irradiated pollen and 22 from 600 Gray irradiated pollen. Within both the 150 and the 300 Gray group, one plant carried two deletions on a single chromosome while in the 600 Gray group seven plants were found each carrying two deletions on different chromosomes. Thus in total 48 deletions were identified and these ranged in size from 30 kb to 3.0 Mb (Table 1). Smaller deletions up to one or a few bp were most certainly induced as a result of NHEJ DSB repair but fell below our detection threshold which lies on average around 200–300 kb. Six of the identified deletions showed total absence of any L*er*-0 reads to the distal sides of their chromosomes suggesting those deletions to be terminal whereas the 42 remaining deletions were interstitial. Remarkably, three of the six apparent terminal deletions were located on the North arm of chromosome 4.

**Table 1:** **The number, average size and largest size of deletions identified per irradiation group**

**Irradiation group**	**150 Gray**	**300 Gray**	**600 Gray**	**Total**
**M2s identified by GA2 genotyping**	14	8	25	47
**M2s after removal of low read individuals**	11	6	22	39
**# M2s carrying two deletions**	1	1	7	9
**# deletions identified**	12	7	29	48
**Average deletion size (kb)**	505	1.324	970	
**Largest deletion (kb)**	2.089	2.649	2.959	

From the 39 M2Δ individuals deemed to carry reliably sized deletions, 16 were selected (Additional file 1), based on size and location of their respective deletions, to be analyzed for changes in RF. Two chromosome 2 deletions carried similarly sized deletions in the same area of their chromosomes (M2Δ-13 and M2Δ-16); four chromosome 5 deletions carried deletions of variable size in the same area (M2Δ-1, M2Δ-5, M2Δ-6 and M2Δ-11) and three chromosome 4 deletions carried two similarly sized and one larger terminal deletion on their North arms (M2Δ-4, M2Δ-7 and M2Δ-15). These M2Δ were selected for recombination analysis in the hope that causal relations between size and position of the deletions and their effects upon RF could be made. The remaining M2Δ were selected for deletions on chromosomes not yet included in the set and for those that carried relatively large deletions as we expected that larger deletions would have larger effects.

A representation of heterozygous and homozygous SNP presence over all chromosomes in these lines was visualized by Integrated Genome Viewer (IGV) in Additional file 1. This table also details size and position of regions of LOH, intervals with near 0.5 Col-0 allele frequency and intervals showing RF less than 1 cM/Mb. Within the 16 selected individuals a total of 20 chromosomal deletions were present; additional data from the BC1 genotyping led us to infer the presence of 15 putative inversions.

On six chromosomes within the 16 M2Δ mutants, RF was zero over two intervals covering 3 to 5 Mb while Col-0 allele frequencies remained at wild-type levels. These cases can be best explained by the presence of large chromosomal inversions in these areas. Also, in nine separate cases RF within a single interval dropped below 1 cM/Mb while leaving Col-0 allele frequencies unaffected. The only heterochromatic deletion that was transmitted through meiosis, as shown by the Col-0 allele frequencies at those positions remaining at 0.25, was located in the pericentromeric region of chromosome 5 (Additional file 2: V-A e, f). Therefore we inferred also these nine smaller instances to be inversions. Similar to chromosomal deletions these putative inversions showed zero RF at the inversion site and an increase in RF within a single interval elsewhere, thus maintaining chromosome-wide RF.

**Figure 2:**
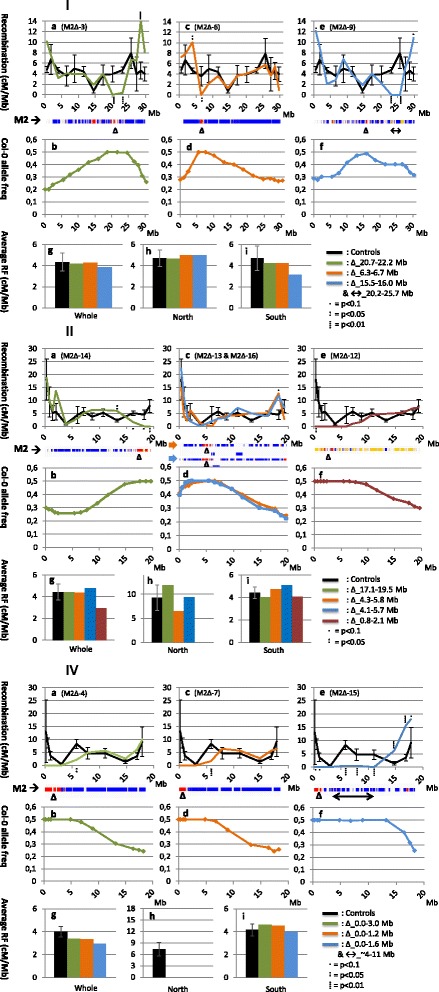
**Recombination frequencies and allele frequencies of Arabidopsis chromosomes (I, II and IV) carrying large chromosomal deletions or putative inversions.** Sections a, c and e show RF over the length of the respective deletion (Δ) or putative inversion (↔) chromosomes (M2→). In the M2 chromosome bars blue sections indicate heterozygous presence of SNPs while red sections show regions of LOH indicating the presence of deletions. Sections b, d and f show col-0 allele frequency over this same length of chromosome. Sections g, h and I show average recombination frequency over the whole chromosomes, their North and South arms. The legends specify the positions of identified deletions and/or inferred putative inversions carried on the respective chromosomes. Black graphs and bars represent data from 3 independent control individuals. Other colors refer to data collected from individual aberrant chromosomes. To be noted in these figures are: zero RF at positions of deletions or putative inversions; upregulations of RF up to 300% of WT in affected chromosomes; Col-0 allele frequency at 0,5 at sites of deletions; variable averages of RF in North and South arms of chromosomes while Whole chromosome RF is mostly comparable to control levels; variable positioning of upregulation in respect to aberration and absence of effects in chromosome 2 and 4 North arm deletions.

BC1 generations (90 individuals) of the selected 16 M2Δs and those of three control individuals were genotyped for 66 SNP markers spread over the genome at ~15-20 cM intervals. In all 20 deletion chromosomes examined except one, Col-0 allele frequency in the BC generation was at 0.5% at the deletion site (Figure 2 b, d, f and Additional file 2: b, d, f). Col-0 allele frequencies gradually drop back to 0.25 as more recombination events take place with distance to the deletion. This shows that of chromosomes that have incurred substantial deletions, only those parts that have recombined with the non-deletion chromosome can be transmitted to the next generation whereas those chromosomes that are missing substantial amounts of DNA are not found in the backcross. However, one exception to this rule was observed in M2Δ-9 where a region of LOH (n_snp_ = 14) measuring 0.3 Mb was present adjacent to the centromere (11.4-11.7 Mb) of chromosome 5 (Additional file 2: V-A e, f). While we do not have data on the BC1 genotype composition at this location, the Col-0 allele frequency of chromosome 5 in the backcross of this individual was ~0.25 at all measured locations. This observation suggests that the absence of this 0.3 Mb stretch of DNA did not inhibit transmission of this chromosome to the next generation. We propose that the vicinity to the centromere of this relatively small deletion may have masked it from selection mechanisms.

### Crossover homeostasis is maintained at wildtype levels in deletion or putative inversion mutants

Recombination frequencies of chromosome deletion lines were compared to three control individuals that were identically handled except for the irradiation treatment were analyzed for RF values on all chromosomes, resulting in the black dataset in Figure 2 and Additional file 2. These data were compared to and do not substantially deviate from published data on male recombination frequencies in Col-0/L*er*-0 hybrids [4].

In the majority of deletion and putative inversion lines examined, the total number of COs was maintained at wild-type frequencies (Figure 2 g and Additional file 2: g) which lay around 1.6 COs per chromosome or 4 cM/Mb in Arabidopsis. Crossover homeostasis was found to be maintained over entire mutant chromosomes while between North and South arms RF was found to vary, depending on size and position of the mutation (Figure 2 h, i and Additional file 2: h, i). Exceptions were found in three independent cases concerning the North arms of chromosomes 2 and 4 where deletions and putative inversions do not induce an upregulation of RF in their respective South arms (Figure 2-II e, h, i and Additional file 2: IV a, c, h, i). A third chromosome 4 North arm mutant did show an increase in RF on its South arm (Additional file 2: IV e, h, i), however this mutant is recombinationally silenced up unto 11 Mb on its South arm while no second deletion is present here. We infer the silencing to result from a large pericentromeric inversion and it is this putative inversion that induced the upregulation of RF on the telomeric side of this arm. The observed RF of 0.5 cM/Mb over two intervals resulted from a singleton in one plant. While inspection of the raw data file showed this singleton to be clearly distinct from all other genotypes at this location, we must assume this instance to have been falsely genotyped.

It is unclear what mechanism would exempt chromosome 2 and 4 North arms from crossover homeostasis and what function this may serve. The North arms of chromosomes 2 and 4 contain the nucleolar organizing regions (NORs). Also they are the shortest arms present among the Arabidopsis chromosomes, measuring about a third the size of the other chromosome arms.

### Maintenance of CO homeostasis brought about through upregulation of RF in single intervals

At sites of deletions or putative inversions no recombination could take place (Figure 2 a, c, e and Additional file 2: a, c, e) as there was no partner to exchange with for the intact chromosome or that partner showed no homology. In most cases the loss of COs at the site of the deletion was compensated for elsewhere on the chromosome. Strikingly, increases in RF were almost exclusively observed to take place in single intervals as opposed to evenly along the chromosome (Figure 2 a, c and Additional file 2: a, c). Rigorous inspection of the raw data files confirmed all significant increases to originate from single COs, not from singletons.

The observed increases were found to be located either proximally to the deletion/putative inversion (Figure 2-I a, c, e) or distally (Figure 2-II a, c) and were also observed on the other side of the centromere (Figure 2-II a). The majority of chromosome 1 compensations took place in close proximity of the deletion and putative inversion sites (Figures 2-I and Additional file 2: I) while chromosome 2 compensations were mostly observed at a distance from the deletion or putative inversion sites (Figure 2-II and Additional file 2: II). Of special significance is Figure 2-II c, as here two independent deletion chromosomes are displayed that carried approximately the same sized deletion in the same area. Although the compensatory upregulations of RF were only significantly different from controls at p < 0.1, these took place in the same interval and were of the same magnitude. For chromosome 1 as well, two chromosomal aberrations in the same chromosome region, one being a deletion (Figure 2-I a) and the other a putative inversion (Figure 2-I e), induced increases in RF of similar strength in intervals at the same distance from the aberration. These observations suggest that there is a causal relation between the site and size of an aberration and the position and extent of compensation in RF.

Chromosomes 3 (Additional file 2: III) and 5 (Additional file 2: V-A and V-B) showed a more varied response to deletion induction and are not specifically discussed here. In these chromosomes as well, crossover numbers were mostly maintained at 1.6 per chromosome although some exceptions were observed. Compensation of RF lost at deletion and putative inversion sites did not exceed standard deviation of controls as clearly as in chromosomes 1, 2 and 4. In the chromosome 3 set however, four out of five mutants shown displayed RF clearly exceeding wild type between 9 and 11 Mb on that chromosome.

## Discussion

We explored the positioning of COs during male meiosis in *Arabidopsis thaliana* mutants carrying large chromosomal deletions and putative inversions generated through pollen γ-irradiation. To this end we analyzed RF for 66 SNP markers in backcrosses of the M2 lines. Previous research into the effect of chromosomal deletions upon CO-positioning on chromosome VI of *Petunia hybrida* showed RF between two tightly linked markers to drastically increase as a function of the size of the induced deletion [15]. In this study we explore this phenomenon further and report both deletions and inversions in Arabidopsis chromosomes to markedly affect RF in specific intervals on the mutant chromosomes while total recombination, over the whole chromosome and genome-wide, typically remains unchanged.

### Irradiation damage

#### Deletions

Gamma rays are widely known to have mutagenic effects due to the damage they cause to genetic material. DSBs are not the most common but are the most important type of damage resulting from ionizing radiation [19]. Ionizing irradiation creates ROS which in turn attack the DNA by creating DSBs [20,21]. Also, a DNA nick can be created by direct absorption of a gamma ray by the sugar phosphate backbone. Subsequent sensitization of the opposing strand may increase the number of DSBs [18]. Unrepaired DSBs leave the DNA vulnerable to exonucleolytic decay. Therefore repair of induced breaks must occur in a timely fashion. In pollen neither the sister-chromatid nor the homologous chromosome is present and therefore repair can only take place through the NHEJ pathway. This blunt-end ligation process with little or no sequence specificity leaves ample opportunity for the omission, inversion or translocation of DNA segments bordering induced DSBs. We genotyped 150 M2 plants and 50 controls for regions of LOH and found 39 of the M2s to harbor a total of 48 chromosomal deletions which varied in size from 30 kb to 3.0 Mb. In all irradiation groups maximum deletion sizes lay between 2 and 3 Mb whereas deletion sizes up to 6 Mb have been reported in other pollen-irradiation experiments [19] and preliminary data from a similar experiment in our lab showed a complete South arm deletion of chromosome 5 (data not shown). This last observation entails a 14 Mb deletion and suggests that the only restriction on the biological viability of deletions would be the presence of a functional centromere. The viability of deletions is further discussed in ‘The fate of induced deletions’ below.

Out of the 48 deletions we analyzed, six showed no marker presence distal of the deletion and appeared to be terminal. However, DNA that does not enjoy the protection of telomeres is rapidly degraded by exonucleases. Upon fertilization these chromosomes may re-acquire telomeres through Break Induced Replication (BIR) [22]. Moreover, for the chromosome 2 and 4 North arms the apparent terminal deletions may in fact be interstitial as no information is available on the structure of the NOR region. Interstitial deletions might arise after repair, when two breaks are induced and NHEJ repair results in the ligation of the two outer pieces of the chromosome while omitting the central part which would subsequently be destroyed by exonucleases. Alternatively, interstitial deletions could arise through the occurrence of a single break where the NHEJ process targeted a micro-homology in an undisturbed part of the chromosome thus ligating the loose end to an ectopic position whilst excising the part of the chromosome between the two micro-homologies [23].

#### Putative inversions

On chromosomes 1, 3, 4 and 5 some deletion mutants displayed a total absence of recombination over several intervals measuring from 2 to over 6 Mb. Col-0 allele frequencies were maintained at 0.25 in all these mutants whereas chromosomal deletions typically yield a 0.5 Col-0 allele frequency in their backcross due to their non-transmissibility [15,24]. The presence of large chromosomal inversions is consistent with the characteristics of these instances and inversions are known to be readily induced as a result of Gamma-irradiation [18]. In addition to these large instances a number of single intervals were found where RF was at or near zero with Col-0 allele frequencies remaining at 0.25 and where apparent compensating increases in RF were observed elsewhere on the subject chromosomes. In all these cases we putatively infer chromosomal inversions to be present although in some cases natural variation in RF may be the cause of the observed phenomenon as single intervals showing near-zero RF have been observed in control individuals. Nevertheless, the objective of our study is to assess the reaction of chromosomes to the presence of regions where no recombination takes place; the exact constitution of these regions is of secondary importance hence these smaller, more equivocal regions are also included in our analysis.

### The fate of induced deletions

Of the 20 deletions analyzed, only one was found to be transmitted from the M2 to its BC. This particular deletion was about 0.3 Mb in size and was located in the pericentromeric region of chromosome 5. All other BCs displayed a Col-0 allele frequency of 0.5 at the deletion site and this frequency dropped off towards 0.25 with distance to the deletion site, showing non-transmission of deletions. Thus, in general, only those parts of a deletion chromosome that have recombined with its intact homologue can be transmitted to the next generation, as has previously been reported for *Petunia hybrida* [15,24]. As recombination with deletion chromosomes did take place and as our induced deletions in mature pollen were delivered to our M2s, selection against chromosomal deletions in all probability takes place between the later stages of meiosis I and the formation of the mature pollen. The observed exception of transmission of a deletion in the peri-centromeric region leads us to suggest that the tight packaging of the chromatin here might hide deletions from cellular selection mechanisms.

As also reported by Yang and coworkers [25], we found the occurrence of aborted seeds to increase with irradiation intensity. Moreover, Yang showed that seed-set, germination and fruitfulness were more negatively influenced when pollen was irradiated earlier in its development, due to dominant lethality caused by chromosomal imbalance or abnormal development of the endosperm [25].

We found some areas of chromosomes within our M2 population not to display any deletions whereas other areas were highly affected. When comparing the sites of induced deletions to gene density plots, there do not appear to be strong correlations between the two. The number of deletion mutants analyzed is too low to draw any strong conclusions on regions of the chromosome or specific genes being ‘undeletable’ but we could hypothesize certain classes of genes to be of such an essential nature that incurred deletions would render the pollen or its offspring either inviable or severely retarded in growth and survival. Pollen tube growth and fertilization are however unlikely to be impaired as large stores of mRNAs needed for these processes are produced prior to anther dehiscence [26].

During the development of the early embryo, communication between cells is essential for the correct formation of cell layers [27,28] as well as embryo axis formation and vascular development [29]. Here, haplo-insufficiance could severely impair the normal developmental processes and result in abortion of the embryo, as is suggested by the increasing number of empty spots as well as shriveled seeds in our M1 siliques as irradiation intensity increased**.** It is in these primary developmental processes that the most stringent selection against the presence of deletions takes place; the survival of M2 plants having past the seedling stage was hardly compromised although many phenotypic aberrations were observed. There were however a number of M2 plants in which the formation of pollen was compromised and where no BC analysis could be made.

### Modulating CO positioning

#### The strength of CO homeostasis differs between and within chromosomes

Seventeen out of 27 displayed mutant chromosomes maintained chromosome-wide RF at wild-type levels. All six chromosome-1 mutants and all three chromosome-2 South-arm mutants maintained wild-type chromosome-wide RF. Six of the 12 mutants that did not maintain wild-type RF concerned chromosome 2 and 4 North arm mutations, where no compensation reaction was induced on the South arm. Of the six remaining mutant chromosomes that did not maintain wild-type chromosome-wide RF, three were found among five chromosome-3 mutants and three were found among eight chromosome-5 mutants. As all Arabidopsis chromosomes share the same intracellular environment these observations suggest differences in chromosomal architecture to effect variations in the implementation of crossover homeostasis between chromosomes and between chromosome arms. Architectural properties of chromosomes have also been suggested to play a role in the non-uniform implementation of interference along the axis of chromosomes and between chromosomes in Arabidopsis [30].

#### Chromosome-2 and −4 North arm deletions and putative inversions do not affect RF in the remainder of the chromosome

Deletions and putative inversions in the North arms of chromosomes 2 and 4 did not result in a compensation reaction whereas such mutations located on the South arm of chromosome 2 generated strong upregulation of RF in specific intervals. However, due to a lack of exploitable markers inside the NOR regions, we have no information on recombination frequencies here and they may well harbor the compensation reactions we would expect. Moreover, we have analyzed only a single chromosome 2 North arm deletion. Although we have explored the effects of several deletions of variable length on the North arm of chromosome 4, the known presence of a large chromosomal inversion between ecotypes Col-0 and L*er*-0 [31] eliminates the possibility for compensation reactions to occur between our induced deletions and the centromere. Therefore it is difficult to draw clear-cut conclusions concerning structural differentiation between these NOR arms and other chromosome arms. Nonetheless, the NOR regions themselves are structurally different from the rest of the genome and these differences have been suggested to be responsible for unexpectedly high levels of interference observed on the NOR arms [30]. Thus despite the lack of definite conclusions we are confident that the NOR arms stand in a special relationship to the recombination machinery that is well worth exploring further.

#### Positions at which COs are allowed appear to be defined by structural features of chromosomes

Both large deletions and inversions completely silence recombination activity at their location because no recombination can occur when there is no template for the partner chromosome. In chromosome-1 mutants as well as chromosome-2 South-arm mutants the loss of COs was compensated for by increases in RF up to 300% of wild-type levels mostly in single intervals. These large responses only occurred in the more distal intervals within a few Mb from the telomeres while intervals proximal to the centromere showed maximum increases just over 150% of control frequencies. Interestingly, in two separate occasions concerning chromosomes 1 and 2, two independent structural aberrations similar in size and extent elicited near-identical responses. These occasions strongly suggest a causal relation between the site and size of the induced aberration and the position and extent of compensation. Although the reaction of pericentromeric regions is less pronounced, the number of chromosome-3 mutants showing an increase in specifically the intervals adjacent to the centromere makes it hard to attribute these observed increases to random variation. The central parts of the large chromosome arms reacted little to induced mutations. Average CO rates in Arabidopsis male meiosis are also found to be high in the proximal parts of chromosomes and very high at both distal ends while relatively low in the intermediary regions [4]. Control of CO formation thus seems to be more stringently imposed on the central areas of chromosome arms than on the proximal and distal regions. This intra-chromosomal differentiation in the regulation of CO distribution most likely results from the chromosome architecture itself. Features like chromatin compaction and mechanical stiffness of chromosomes influence the accessibility of the chromatin to the recombination machinery and as such these factors have been suggested to implement the interference between COs observed in most eukaryotes [32].

## Conclusions

When taken together, these observations show that changes in the physical structure of the chromosome can have large effects on the positioning of its COs. Moreover, different reactions to induced structural changes are observed between and within chromosomes. However, the similarity in reaction of chromosomes carrying similar changes suggests a direct causal relation between induced change and observed reaction.

## Methods

### Plant material

*Arabidopsis thaliana* accessions Columbia (Col-0, N1092), Landsberg *erecta* (L*er*-0, NW20) and the male-sterile L*er*-0:ms1ttg1 (N261) were obtained from the Nottingham Arabidopsis Stock Centre (NASC; www.arabidopsis.info). L*er*-0:ms1ttg1 was maintained as heterozygote and used as the female backcross (BC) parent in a homozygous recessive (−/−) state.

All seeds were sown on a 3:1 mixture of standard soil (Lentse potgrond no. 4) and vermiculite and seedlings were transplanted to individual pots in the four leaf stage. The plants were grown in growth rooms under standard conditions (16 h light, 8 h dark, 40-60% humidity, 20°C). DNA was isolated 6–8 weeks after germination.

### Irradiation treatment and genetic crosses

Whole, flowering plants of *Arabidopsis thaliana* ecotype L*er*-0 were subjected to γ-radiation from a Cobalt^60^ source (Isotron Nederland BV, Ede, the Netherlands) at intensities of 0, 150, 300 and 600 Gray (0, 15, 30 and 60 krad). On the same afternoon, pollen of these individuals was used to fertilize Col-0 plants (Figure 1). All crosses were made between two single individuals. The plants developed their siliques in standard growth room conditions, equal to their previous growth conditions. Siliques were harvested as they were turning yellow. Seeds were dried for two weeks minimum prior to sowing.

For the fertilization with M1 L*er*-0 pollen, unopened Col-0 flowers were carefully trimmed of sepals, petals and anthers. Open L*er*-0 flowers were picked at their bases with a pair of forceps, thus exposing the anthers which were then rubbed against the exposed Col-0 pistils. Fertilized pistils were marked with strings of thin yarn. Backcrossing took place by just rubbing the open M2 flowers against open male-sterile L*er*-0:ms1ttg1 (−/−) flowers. These were also marked with yarn.

From pilot studies we performed and reports by Naito and colleagues [19], we knew that silique length and seed set of the plants fertilized with irradiated pollen as well as germination of both the M2 seeds and the BC1 seeds were substantially diminished with increasing irradiation strength. To compensate for this, the number of crosses of M1 L*er*-0 pollen to Col-0 and M2 pollen to L*er*-0:ms1ttg1 stigmas were increased with increasing irradiation intensity. We aimed to obtain at least 200 viable seeds per cross. At 0 Gray 5 crosses were made while at 600 Gray 15 pistils were fertilized.

### DNA extraction

Two young leaves of 6–8 week old plants were placed in collection microtubes (Qiagen, Hilden, Germany, cat. no. 19560) containing a 3 mm ball bearing. Microtubes were closed with caps (Qiagen, Hilden, Germany, cat no. 19566) and placed in liquid nitrogen. Frozen leaves were ground in a Mixer Mill (Retch MM300, Haan, Germany) for 30 s at 30 Hz and 500 μl extraction buffer (1% N-cetyl-N,N,N-trimethyl-ammonium bromide (CTAB), 100 mM Tris–HCl pH 7.5, 700 mM NaCl, 10 mM EDTA in aqua bidest (Milli-Q, MQ)) at 60°C was added to each collection tube. The tubes were closed, shaken well and incubated for 30 min at 60°C. The samples were cooled on ice for 15 min; 250 μl chloroform/isoamylalcohol (24/1) was added and the tubes were shaken intensely for a few minutes. Samples were placed in a centrifuge (Sigma 4-15C, Osterode am Harz, Germany) at 4000 rpm for 10 min. 250 ml of the supernatant was transferred to new collection tubes (Brand, cat. no. 781525) containing 200 μl isopropanol. The tubes were closed with collection tube caps (Brand cat. no. 781535), the samples were mixed by inverting the tubes several times and the tubes were then centrifuged for 10 min at 4000 rpm. The supernatant was discarded, 70% ethanol was added to the tubes and these were centrifuged for 10 min at 4000 rpm. The supernatant was discarded and the pellet was vacuum dried before dissolving the DNA in 200 μl MQ. Samples were stored at 4°C for short-term storage and at −80°C for long-term storage.

### M2 genotyping

DNA was isolated from about 70 M2 plants of each of the irradiated populations (0, 150, 300 and 600 Gray) using a standard CTAB protocol. Fifty plants per group were subjected to sequence-based genotyping at Keygene N.V. as previously described [33]. In short, total genomic DNA was digested with *Eco*RI and *Mse*I restriction enzymes. Universal *Mse*I adapters were ligated to the *Mse*I restriction site overhangs whilst sample-specific adapters were ligated to the *Eco*RI restriction site overhangs. The *Eco*RI adapters contained a unique 5 nt sample identification tag. Three groups of 70 samples were pooled to make three libraries. Three lanes on an Illumina Genome analyzer II were used for single-end sequencing (72 nt) of these libraries. The raw data were used for deletion detection.

### Deletion detection

Deletions were detected in Col-0/L*er*-0 M2 plants through the genotyping of heterozygous SNPs. Large deletions in our irradiated L*er*-0 genome would show up as areas of loss of heterozygosity (LOH) where only the Col-0 genotype would be present over multiple markers. When such areas consist of three or more consecutive SNP-bearing reads covered by six or more reads, these are defined as being deletions.

The data resulting from the Illumina genotyping was analyzed in collaboration with the Netherlands Bioinformatics Centre (NBIC). The online available Arabidopsis genome sequence version TAIR10 (www.Arabidopsis.org) was cut *in silico* at the *Eco*RI restriction site and resulted in the identification of 37,057 theoretical cut sites. As sequencing was performed both on the plus and the minus strands this resulted in 74,114 theoretical target sites. The 72 bp short reads were aligned against the Col-0 reference genome (tair10.fasta; www.arabidopsis.org) using Burrows-Wheeler Aligner (BWA) on default settings. Of the sequenced reads, 90% mapped back to the reference sequence; 20%-30% of the reads mapped to the chloroplast genome. Read coverage for each theoretical region was determined using bedtools. About 14% of the theoretical targeted regions were covered ≥ 5X, while about 12% of the covered regions have both flanking regions of the cut site covered. SNPs were called as variants using samtools mpileup|varscan mpileup2snp. Alignments with mapping quality <30 (mapQ < 30) were skipped to down-weight the contribution of the low mapping quality reads. Variants were called when there was at least 5X read depth at a each position and at least 2X variant supporting reads to make a call. As the sites covered in control individuals varied greatly, a ‘Majority Vote’ strategy was adopted wherein at least 10 out of 47 control plants must consistently display the same genotype for that genotype to be accepted as genuine. This resulted in a list of heterozygous SNP sites (markers) for non-radiated M1 (See Additional file 3). When compared to previously published SNPs [34], about a quarter of our SNPs do not coincide. However, the SNPs that do not coincide are randomly distributed and due to our minimum detection threshold for deletions of three subsequent, read independent SNPs, these disparities are not expected to influence our data.

For each radiated M1 plant, the following information on each marker site was extracted: (a) genotype; (b) number of reference base supporting reads/number of variant base supporting reads and (c) read depth. For each ’irradiated’ M1 plant, consecutive marker sites with loss of heterozygosity (LOH) were identified to infer a deletion. An inferred deletion event has to fulfill the following criteria: (1) at least 3 consecutive markers with LOH; (2) at least 6X read depth on each of the consecutive LOH marker sites (*i.e.* to distinguish no call due to no coverage *vs.* no call due to LOH during the variant calling step); (3) at least 3 distinct target intervals (*i.e.* down-weight the contribution of LOH markers from the same target interval).

### Discontinuous deletions

Some of the originally defined deletion mutants exhibited the presence of ‘internal’ L*er*-0 reads within a ‘Col-0 only’ section, indicating the occurrence of discontinuous deletions. In one out of the twelve 150 Gray deletions, two out of the seven 300 Gray deletions and six out of the thirty-four 600 Gray deletions this phenomenon was found. At all locations except one this concerns a single read carrying one to three SNPs; at the other location two overlapping reads are involved.

In two separate chromosome 2 M2Δ individuals, discontinuous deletions overlapped with each other. Exactly the same situation was found for chromosome 5. When comparing L*er*-0 SNP locations between the individuals in both sets (See Additional file 4), the majority of internal L*er*-0 reads map to identical locations in both partners of each set. The data from chromosome 5 show the two adjoining SNPs from overlapping reads being similarly covered in both M2Δs. The congruency of mapping within both sets suggests either the unlikely event that those parts of chromosomes have a strong physical disposition to the effects of gamma rays, generating DSBs at specific positions in close proximity to each other, or, more likely, that these reads are derived from sequences elsewhere in the genome and were mapped erroneously to most if not all of these locations. This was found to be so for the instances at 9570955 bp and 9570964 bp on chromosome 5 which were found to correspond to a known TE-induced deletion in *Ler*-0: AMU-5-162 [35].

### Inversion detection

Chromosomal inversions are known to be readily induced as a result of gamma-irradiation. Inversions were not detectable in the sequencing procedure as no assembly of the sequencing data was made. However, data from BC genotyping showed a number of chromosomes to be devoid of COs over one or more intervals while Col-0 allele frequencies remained unchanged and while losses in RF were compensated for on the chromosome. We therefore putatively inferred these regions to represent inversions.

### BC genotyping

All M2 individuals were backcrossed to L*er*-0:ms1ttg1. Using a standard CTAB protocol, DNA was isolated from 92 BC offspring of each of the 16 selected deletion individuals and of three control individuals. Sixty-six SNPs (see Additional file 5) were selected from a list of SNPs previously published [4] to cover the five chromosomes at approximately 15–20 cM distances from each other with an increase in density towards the telomeres. Development and validation of primers for SNP detection and the subsequent genotyping of BC generations using KASP essays was performed by LGC genomics.

### Recombination analysis

As BC generations were generated by crossing the M2 individuals (Col-0/L*er*-0) to L*er*-0:ms1ttg −/−, on average the BCs will consist of a 3:1 proportion of L*er*-0:Col-0 genotypes. Col-0 allele frequencies are thus 0.25 in wild-type situation while they are 0.5 in the case of a deletion being present as no L*er*-0 chromosome was present at the deletion site in the M2.

Genotypes of the BC individuals were compared between all neighbouring markers that gave reliable results. Individuals containing two or more adjoining markers of not determined genotype were removed from the data. Cases of single undetermined markers were matched to their neighbours if both neighbours were of the same genotype (*i.e.* Col:L*er*/NA/Col:L*er*) or set to the average of the remaining population when the neighbours were not of the same genotype (*i.e.* Col:L*er*/NA/Col:Col). Missing data in the first and last marker of the chromosomes were set to their neighbour, generating a slight underestimation of RF in some of the most distal intervals. A few individuals where the genotype switched between every adjoining marker for more than three markers were removed. On chromosome 3 two markers (PERL_466437_L*er*; 6504230 bp) and (PERL_502764_L*er*; 9994092 bp) gave unreliable results in five respectively four BCs. The four fall within the five. In all subject BCs, the entire marker was removed from the dataset and RF was measured between the two adjoining SNPs.

The total number of COs between two markers was divided by the number of individuals in the BC and the result was divided by the length of the interval between the two markers to generate the RF in cM/Mb. Average cM was calculated by dividing the cumulative cM by the distance between the first and last marker of the chromosome. The frequencies of Col-0 allele presence were calculated by summing the number of heterozygote calls at each marker and dividing this by the number of individuals in the BC.

RF in all intervals was compared to pooled controls using a Student’s T-test including Benjamini correction. Significant differences from controls of p < 0.1, p < 0.05 and p < 0.01 are displayed per interval in Figure 2 and in Additional file 1. For all chromosomes except chromosome 5 variation between control individuals fell well within expected levels of variation p < 0.1. For one interval of chromosome 5 the controls were significantly different from each other (p < 0.01). Chromosome 5 was therefore excluded from our analysis.

## Additional files

Additional file 1:
**Overview of the chromosome constitution of selected deletion mutants and of their behavior in the backcross, leading to the type of aberration inferred.** Here are listed the analyzed chromosome aberration mutants and control plants (column A), regions of LOH visualized by IGV (column B), regions of LOH above our threshold levels leading us to infer deletions (column C), chromosome sections where BC Col-0 allele frequencies at or near 0.5 (column D), chromosome sections where RF falls below 1% (column E), and the type of aberration we infer to be present based on the data listed (column F). Specifics are listed below the tables for ease of access. Additional file 2:
**Recombination frequencies and allele frequencies of Arabidopsis chromosomes (I, IV, V and III) carrying large chromosomal deletions or putative inversions.** Sections a, c and e show RF over the length of the respective deletion (Δ) or putative inversion (↔) chromosomes (M2→). In the M2 chromosome bars blue sections indicate heterozygous presence of SNPs while red sections show regions of LOH indicating the presence of deletions. Sections b, d and f show col-0 allele frequency over this same length of chromosome. Sections g, h and i show average recombination frequency over the whole chromosomes, their North and South arms. The legends specify the positions of identified deletions and/or inferred putative inversions carried on the respective chromosomes. Black graphs and bars represent data from 3 independent control individuals. Other colors refer to data collected from individual aberrant chromosomes. To be noted in these figures are: zero RF at positions of deletions or putative inversions; upregulations of RF up to 300% of WT in affected chromosomes; Col-0 allele frequency at 0,5 at sites of deletions; variable averages of RF in North and South arms of chromosomes while Whole chromosome RF is mostly comparable to control levels; variable positioning of upregulation in respect to aberration and absence of effects in response to chromosome 2 and 4 North arm deletions. Additional file 3:
**List of SNP markers used for deletion detection.** These were selected by means of a Majority vote strategy as explained in Materials and Methods, section ‘Deletion detection’. Additional file 4:
**A comparison of two sets of independent discontinuous deletions covering the same areas on chromosomes 2 and 5 respectively.** These comparisons show that the majority of internal L*er*-0 mappings in these regions occur at the same positions in different individuals, suggesting that these reads originate from other places in the genome and that these are mapped here erroneously. Therefore all discontinuous deletions are treated as continuous deletions in our analyses. Blue highlighted areas fall outside our defined deletion areas whereas red highlighted areas are chromosomal deletions as defined in Materials and Methods, section ‘Deletion detection’. Additional file 5:
**SNP list as used for BC genotyping using KASP essays.** This list has been adapted from [4].
