# Supplementary figures and images for: Modulating crossover positioning by introducing large structural changes in chromosomes
Source: BMC Genomics. 2015 Feb 15;16(1):89. doi: 10.1186/s12864-015-1276-z (PMC4359564; doi:10.1186/s12864-015-1276-z)

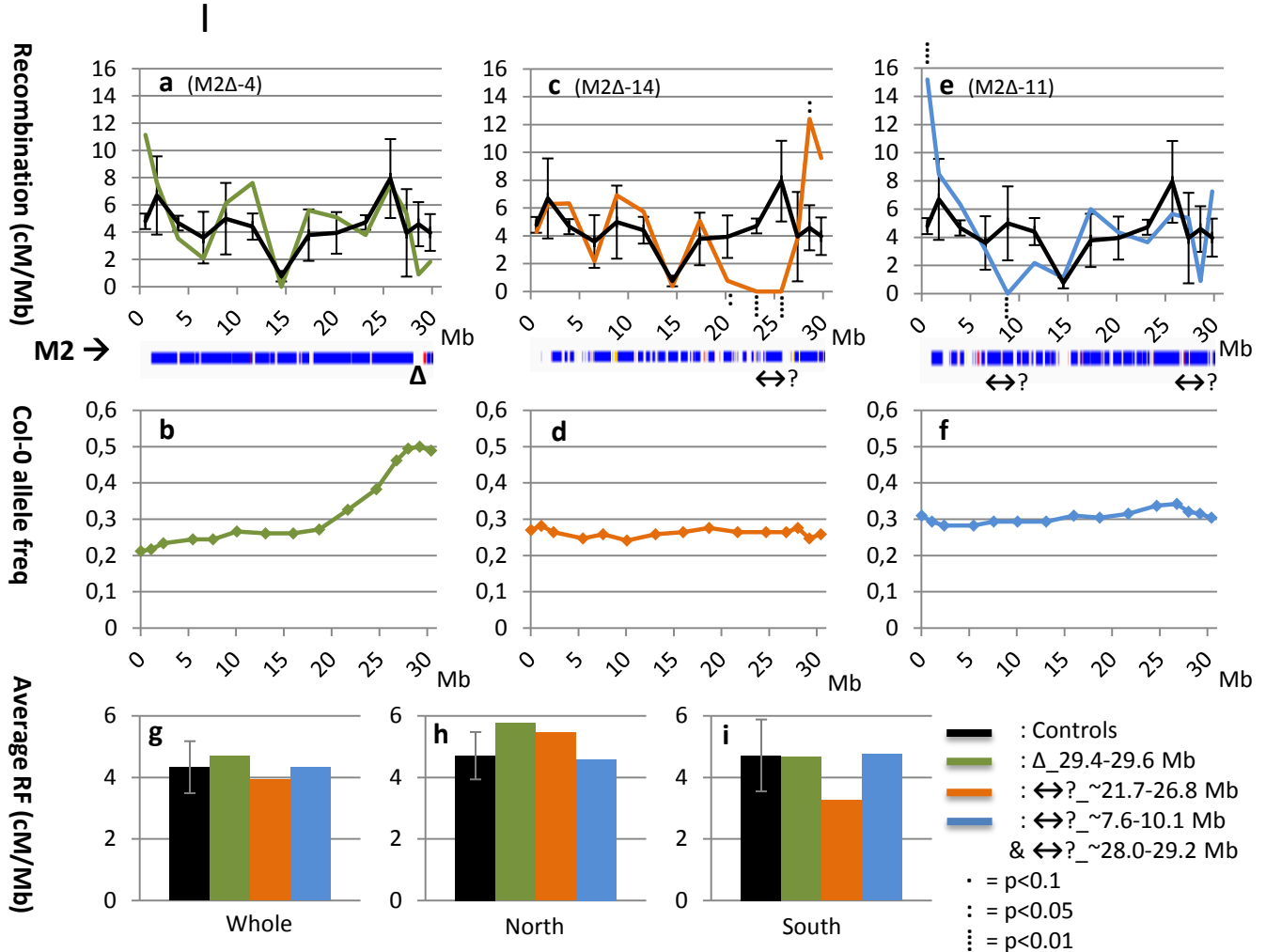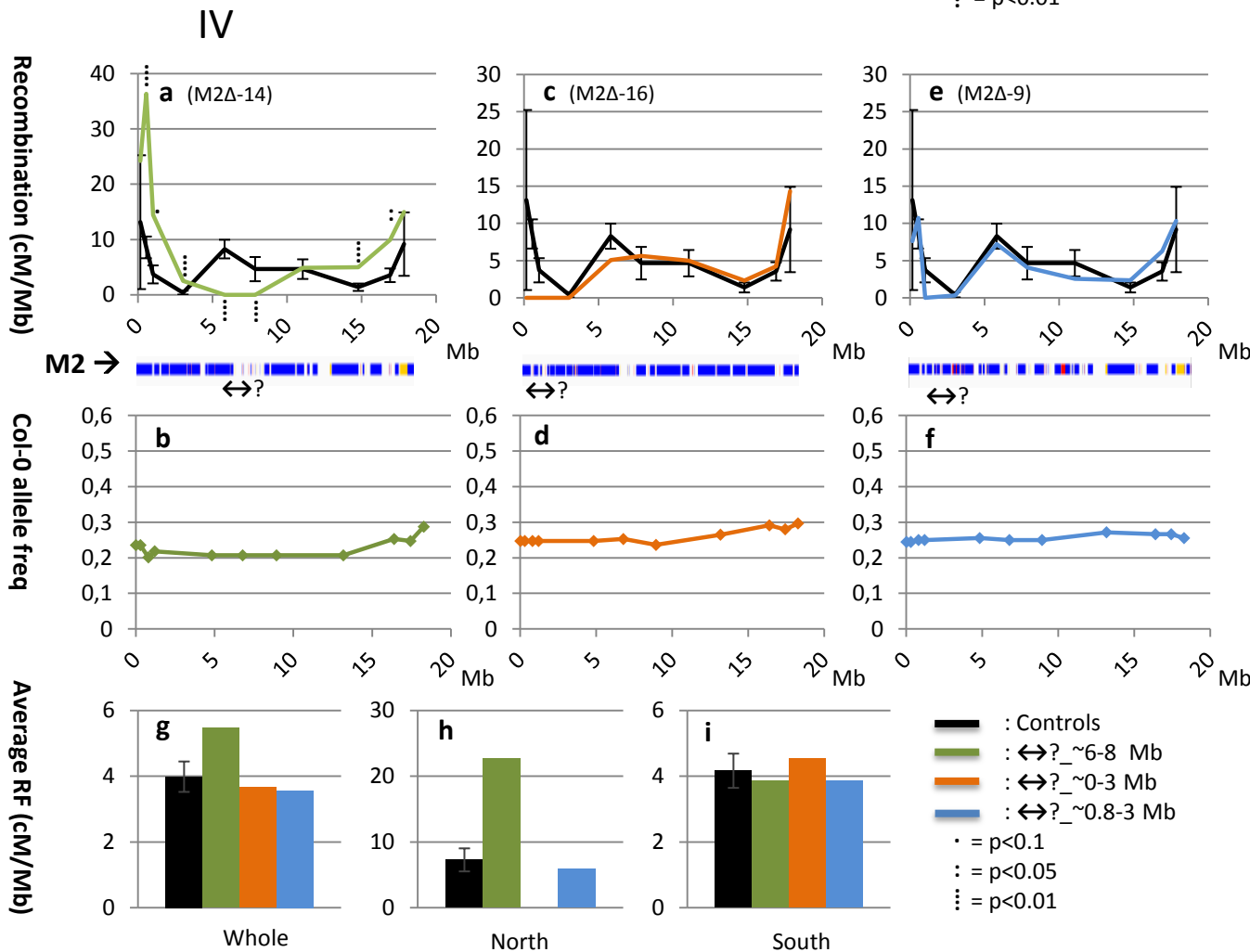

# V-A

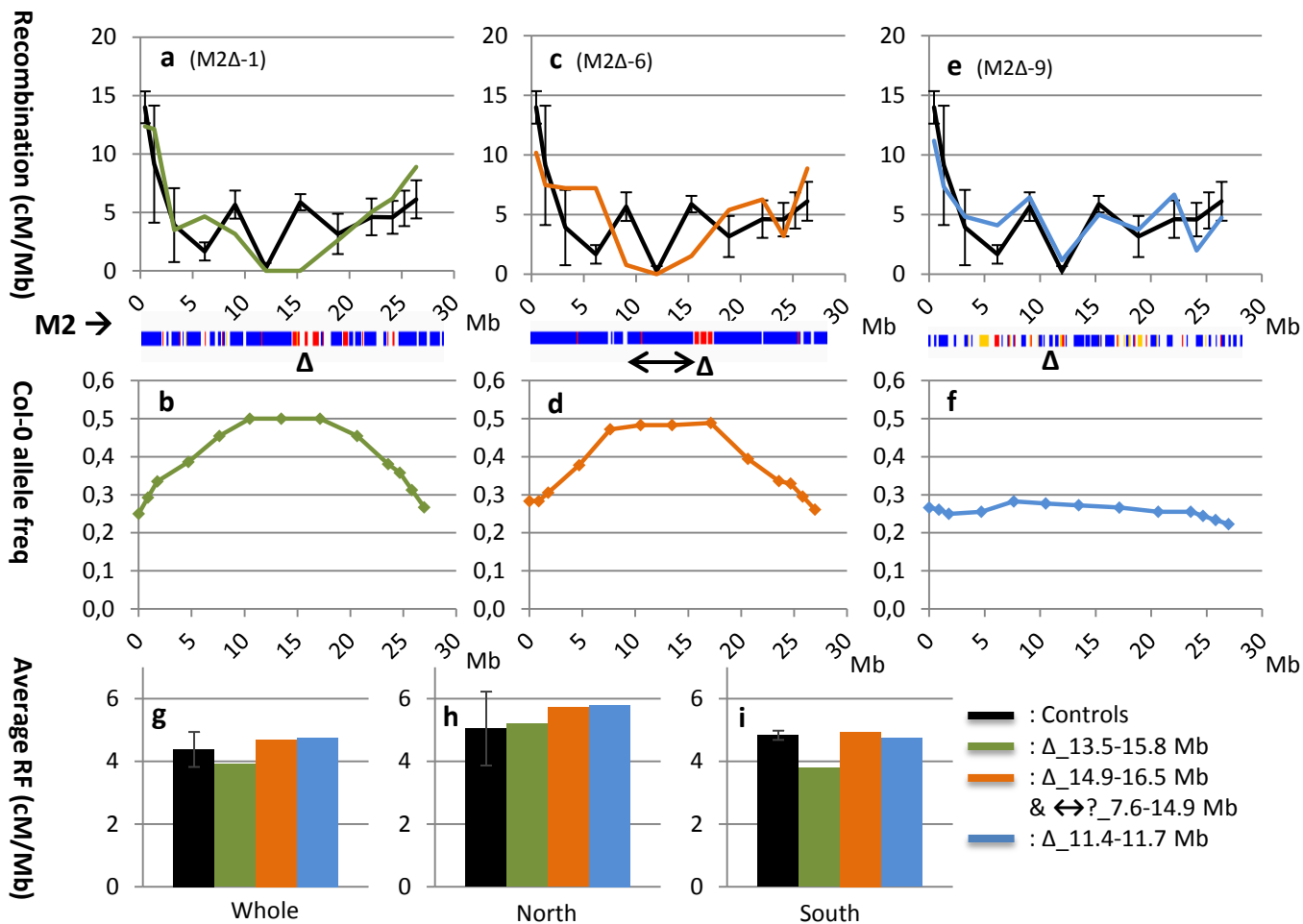

# V-B

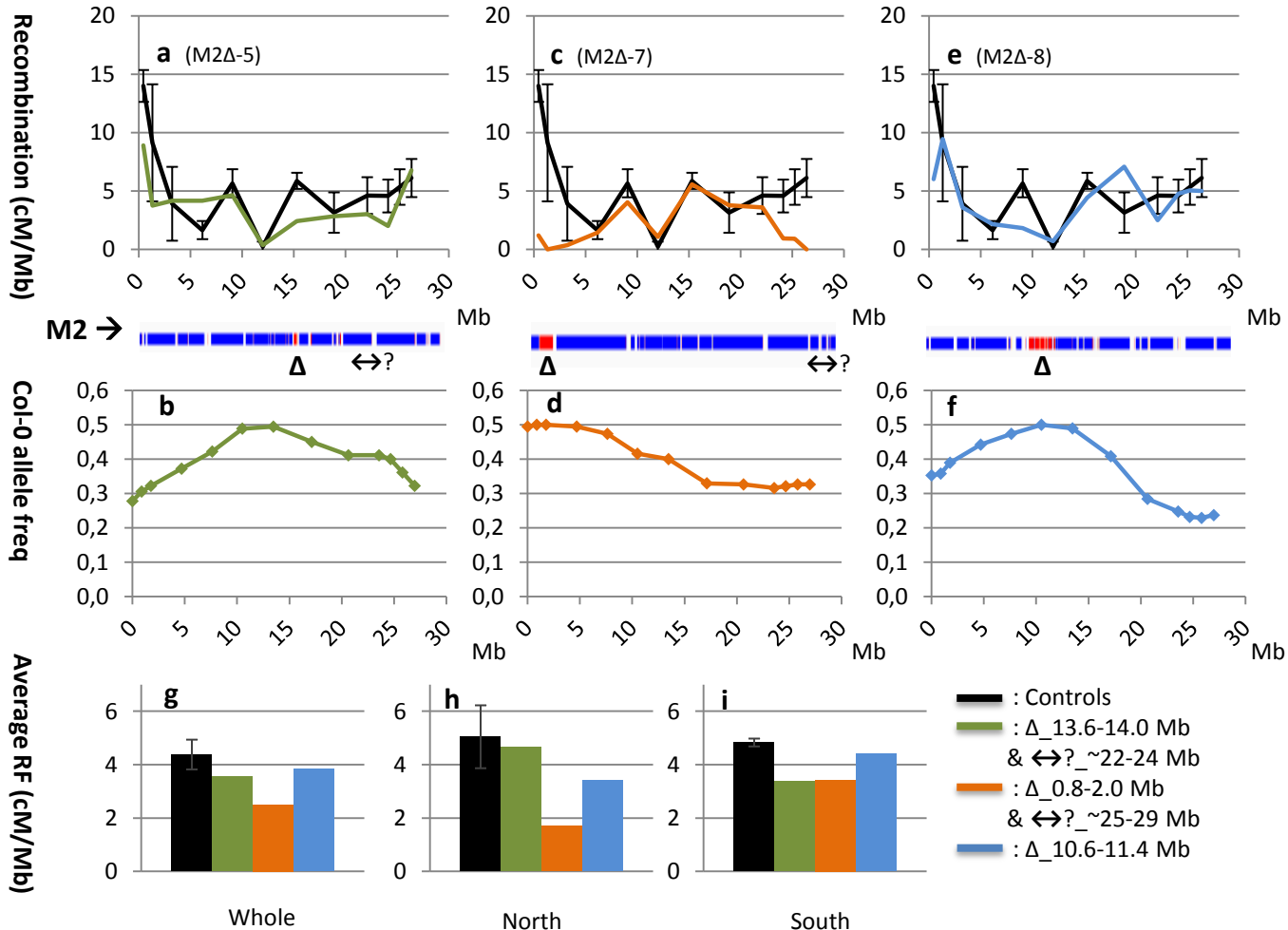

III

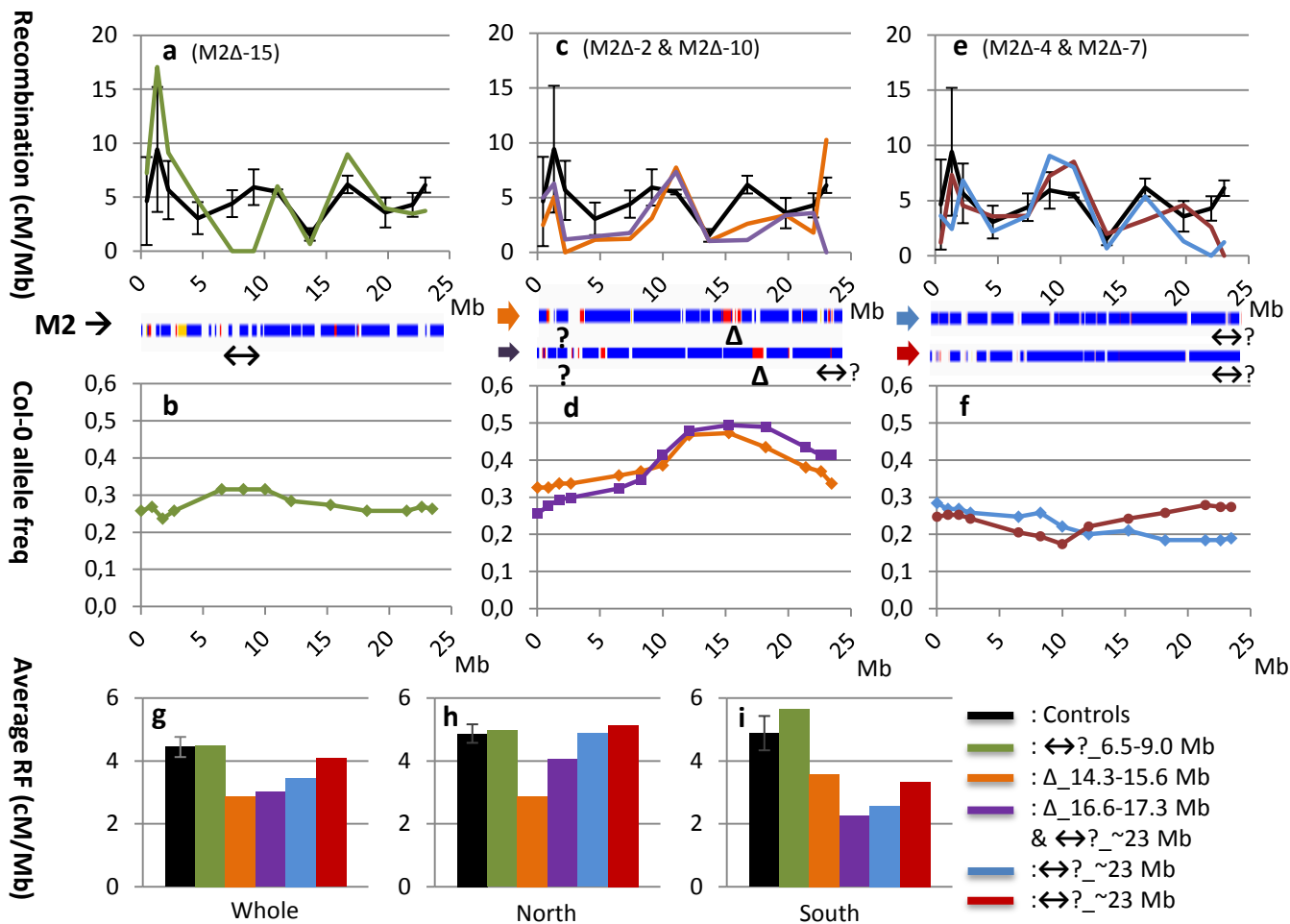

Supplement: Additional file 2: — Recombination frequencies and allele frequencies of Arabidopsis chromosomes (I, IV, V and III) carrying large chromosomal deletions or putative inversions. Sections a, c and e show RF over the length of the respective deletion (Δ) or putative inversion (↔) chromosomes (M2→). In the M2 chromosome bars blue sections indicate heterozygous presence of SNPs while red sections show regions of LOH indicating the presence of deletions. Sections b, d and f show col-0 allele frequency over this same length of chromosome. Sections g, h and i show average recombination frequency over the whole chromosomes, their North and South arms. The legends specify the positions of identified deletions and/or inferred putative inversions carried on the respective chromosomes. Black graphs and bars represent data from 3 independent control individuals. Other colors refer to data collected from individual aberrant chromosomes. To be noted in these figures are: zero RF at positions of deletions or putative inversions; upregulations of RF up to 300% of WT in affected chromosomes; Col-0 allele frequency at 0,5 at sites of deletions; variable averages of RF in North and South arms of chromosomes while Whole chromosome RF is mostly comparable to control levels; variable positioning of upregulation in respect to aberration and absence of effects in response to chromosome 2 and 4 North arm deletions. [file 12864_2015_1276_MOESM2_ESM.pdf]
